# Utility of Minimally Invasive Technology for Inguinal Lymph Node Dissection in Penile Cancer

**DOI:** 10.3390/jcm9082501

**Published:** 2020-08-03

**Authors:** Reza Nabavizadeh, Benjamin Petrinec, Andrea Necchi, Igor Tsaur, Maarten Albersen, Viraj Master

**Affiliations:** 1 Department of Urology, Emory University School of Medicine, Atlanta, GA 30322, USA; bpetrinec@neomed.edu (B.P.); vmaster@emory.edu (V.M.); 2 Department of Medical Oncology, Fondazione IRCCS Istituto Nazionale dei Tumori, 20133 Milan, Italy; Andrea.Necchi@istitutotumori.mi.it; 3 Department of Urology and Pediatric Urology, University Medicine Mainz, 55131 Mainz, Germany; Prof.Dr.med.Igor.Tsaur@unimedizin-mainz.de; 4 Department of Urology, University Hospitals Leuven, 3000 Leuven, Belgium; maarten.albersen@uzleuven.be

**Keywords:** penile cancer, inguinal lymph nodes, inguinal lymphadenectomy, minimally invasive, robotic, endoscopic, video-assisted

## Abstract

Our aim is to review the benefits as well as techniques, surgical outcomes, and complications of minimally invasive inguinal lymph node dissection (ILND) for penile cancer. The PubMed, Wiley Online Library, and Science Direct databases were reviewed in March 2020 for relevant studies limited to those published in English and within 2000–2020. Thirty-one articles describing minimally invasive ILND were identified for review. ILND has an important role in both staging and treatment of penile cancer. Minimally invasive technologies have been utilized to perform ILND in penile cancer patients with non-palpable inguinal lymph nodes and intermediate to high-risk primary tumors or patients with unilateral palpable non-fixed inguinal lymph nodes measuring less than 4 cm, including videoscopic endoscopic inguinal lymphadenectomy (VEIL) and robotic videoscopic endoscopic inguinal lymphadenectomy (RVEIL). Current data suggest that VEIL and RVEIL are feasible and safe with minimal intra-operative complications. Perhaps the strongest appeal for the use of minimally-invasive approaches is their faster post-operative recovery and less post-operative complications. As a result, patients can tolerate this procedure better and surgeons can offer surgery to patients who otherwise would not be a candidate or personally willing to undergo surgery. When compared to open technique, VEIL and RVEIL have similar dissected nodal count, a surrogate metric for oncological adequacy, and a none-inferior inguinal recurrence rate. Larger randomized studies are encouraged to investigate long-term outcome and survival rates using these minimally-invasive techniques for ILND.

## 1. Introduction

**Figure 1:**
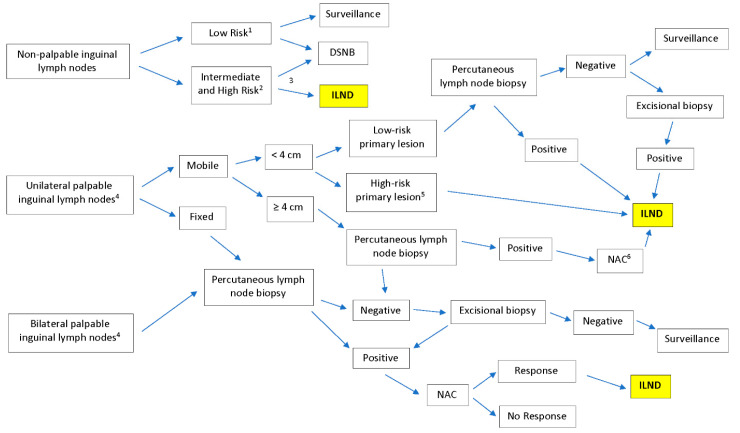
Summary of National Comprehensive Cancer Network (NCCN) guidelines for treatment of penile cancer and the role of inguinal lymph node dissection (ILND). DSNB, dynamic sentinel lymph node biopsy; NAC, neoadjuvant chemotherapy. ^1^ Tis, Ta, and T1a disease; ^2^ T1b and T2 or greater disease; ^3^ Imaging including abdominal/pelvis computed tomography (CT) or magnetic resonance imaging (MRI), with contrast and chest imaging (CT or X-ray); ^4^ Imaging modalities include abdominal/pelvis CT or MRI, with contrast and chest imaging (CT or X-ray) for palpable lesions which provide information that guides therapeutic steps shown in the figure; ^5^ T1, high-grade, lymphovascular invasion, >50% poorly differentiated; ^6^ Patients ineligible for cisplatin-based chemotherapy can move straight to ILND. ILND was highlighted for the purpose of bringing attention to the procedure, as it is a complex chart.

Inguinal lymph node dissection (ILND) is an important component of staging and treatment of penile cancer as well as some other malignancies that can metastasize to this area of the body [1,2,3]. In penile cancer, the extent of inguinal lymph node involvement is one of the strongest prognostic indicators of long-term survival [4]. In addition to excision of the primary tumor, National Comprehensive Cancer Network (NCCN) guidelines recommended ILND in penile cancer patients within multiple different clinical scenarios [2]. Figure 1 summarized the work up and indication for ILND. In addition to the importance of accurate staging, early treatment of inguinal lymph node involvement is associated with improved survival [5,6].

However, ILND historically has been associated with high morbidity and long convalescence [7]. The high rates of post-operative complications make this surgery unappealing to patients and even providers, despite the significant oncological benefits. In this review, we discuss minimally invasive approaches to ILND including their complications and oncological outcomes. Although direct comparisons between open ILND and minimally invasive techniques are sparse in the literature, landmark studies of open ILND are discussed in order to place the reviewed outcomes of minimally invasive techniques in context. 

## 2. Materials and Methods

The PubMed, Wiley Online Library, and Science Direct databases were reviewed in March 2020 for relevant studies limited to those published in English and within 2000–2020. The search included the keywords “Inguinal Lymph Node Dissection”, “ILND”, or “Inguinal Lymphadenectomy” and “Videoscopic”, “Videoendoscopic”, “Robotic”, Robot-assisted”, “VEIL”, “VIL”, “RVEIL”, “RVIL”, or “Minimally-invasive”. Forty-seven articles on ILND regardless of the underlying malignancy were included for review. Thirty-one of these articles pertained to penile cancer. For surgical steps and post-operative recovery, series were reviewed regardless of underlying malignancy. In the discussion concerning oncologic outcomes and survival, we included series for penile cancer only. Table A1 summarizes the articles discussed throughout this paper. Articles were excluded if they discussed obsolete or less commonly practiced guidelines, techniques, or were irrelevant, or had no full-text reference. Articles were also excluded if they were technique videos/descriptions without reporting patient characteristics, morbidity, or outcome.

## 3. Results

### 3.1. Inguinal Lymph Node Dissection

Open ILND is performed via an open incision, usually 2 cm below the inguinal ligament, and is associated with a high rate of complications such as skin edge necrosis, wound dehiscence, infection, lymphocele, lymphorrhea, femoral vessel and femoral nerve injury, deep vein thrombosis, and chronic extremity lymphedema. Reported total complication rates range from 50–90% with a significant impact on quality of life, potentially limiting utilization of recommended ILND for oncologic indications [7,8,9]. A retrospective study using the National Cancer Database from 2004 to 2014 showed that only 25.3% of patients underwent ILND and/or the dynamic sentinel node biopsy (DSNB) [10]. In particular, nonacademic institutions were less likely to adhere to recommendations for surgical staging of inguinal lymph nodes [10].

DSNB offers a method to sample inguinal lymph nodes potentially avoiding ILND and its associated morbidity if the result is negative. However, DSNB is a technically challenging procedure. Historically, this technique has been shown to have false-negative rates as high as 25% [11]. However, with modification to the technique, the sensitivity has improved to over 90% [12]. Incorporation of DSNB into treatment should be limited to high-volume tertiary-care centers where at least 20 procedures are done per year [13]. Additionally, performing DSNB is currently considered not standard of care in patients with palpable lymph nodes.

To diminish the morbidity of the ILND, two different surgical approaches, and sometimes a combination of both, were suggested. One is to modify the dissection template and the other is to use minimally invasive technology to perform the surgery. 

### 3.2. Modifications of ILND Templates

The anatomical landmarks for standard ILND template are the inguinal ligament superiorly, the adductor longus muscle medially, sartorius muscle laterally, and the apex of the femoral triangle inferiorly. The dissection sometimes involves ligation and excision of the proximal greater saphenous vein and complete dissection of the femoral vessels within the femoral triangle. Sartorius muscle can be mobilized for coverage of the femoral vessels.

There are multiple different modifications proposed to the above template. A modified dissection template proposed by Catalona includes preserving the greater saphenous vein and limiting the dissection to the lateral edge of the femoral artery and superficial to the fossa ovalis [14]. Tsaur et al. evaluated the complication rates of their limited template dissection [15]. This dissection further modified the template described by Catalona. For their dissection, the cranial border was formed by a line drawn between the anterior superior iliac spine and the pubic tubercle. Lateral border was a 20 cm perpendicular line drawn inferiorly from the anterior superior iliac spine. Medial border was marked with a perpendicular 18 cm line drawn from the pubic tubercle down. The caudal border was formed by connecting the inferior part of the lines that marked the medial and lateral borders [14,15]. They performed 57 of these inguinal lymph node dissections. They reported a total post-operative complication rate of 54.4% and 26.3% of dissections experienced a major complication. Major complications included 8% wound infection requiring intravenous antibiotics, 7% lymphocele requiring intervention, 1.8% deep vein thrombosis, and 15.8% wound re-exploration due to formation of abscesses, hematomas, infected lymphoceles, or poor wound healing. In addition, 15.8% of dissections experienced mild or moderate leg edema, 7% developed a wound infection (not requiring intravenous antibiotics), 3.5% developed seroma, and 1.8% experienced paresthesia [15].

The rates of complications are variable among different studies in part due to differences in methodologies and definitions. Overall, multiple studies have described the use of different modified open ILND templates which seems to result in improved, but still persistently high, complication rates [7,14,15,16,17,18]. 

### 3.3. Minimally Invasive Approaches

Minimally invasive technologies, including laparoscopic and robotic-assisted approaches, have been utilized to perform ILND in an attempt to decrease morbidity without sacrificing oncologic outcomes. Minimally invasive techniques have primarily been described in penile cancer patients with non-palpable or small palpable lymphadenopathy. However, the use of these techniques in patients with significant palpable inguinal lymphadenopathy as well as following treatment with neoadjuvant therapy is also reported [19,20,21]. Here, we discuss utilization of these approaches including their surgical techniques, peri-operative results and complications, and oncological outcomes. It would be interesting to stratify patients based on the stage of their disease and evaluate the results and complications accordingly. However, current limited data and inconsistent reporting of staging in some case series make this evaluation impractical.

### 3.4. Videoscopic Endoscopic Inguinal Lymphadenectomy (VEIL) Technique

**Figure 2:**
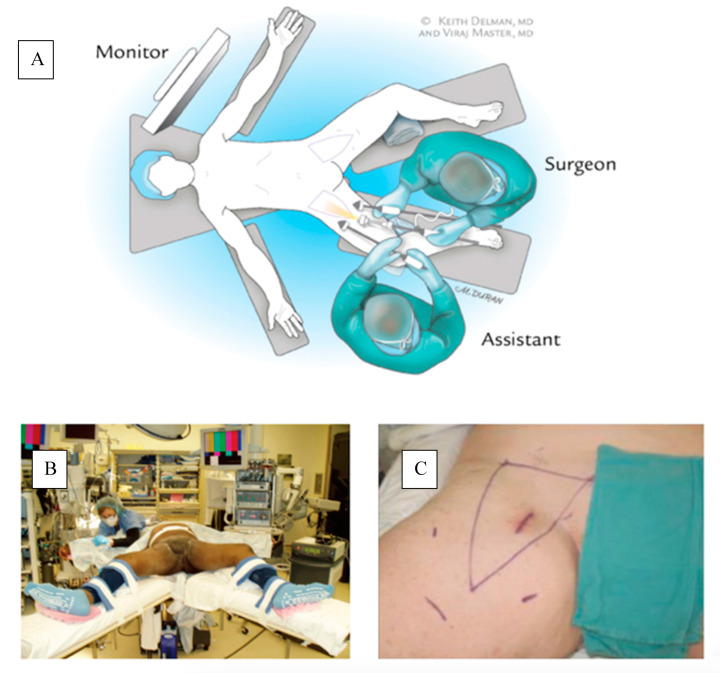
(**a**) Patient and surgeons’ position during the operation. (**b**) Patient is placed on split leg table. (**c**) Landmarks are marked including port sites and femoral triangle. Copyright owned by Master et al.

After induction of general anesthesia, patients are placed in supine position with their legs externally rotated and abducted on a split-leg table. Surgical landmarks including the femoral triangle are marked (Figure 2). The boundaries of dissection are similar to open ILND. The surgeon stands on the medial and the assistant on the lateral side of the operative limb (Figure 2). 

**Figure 3:**
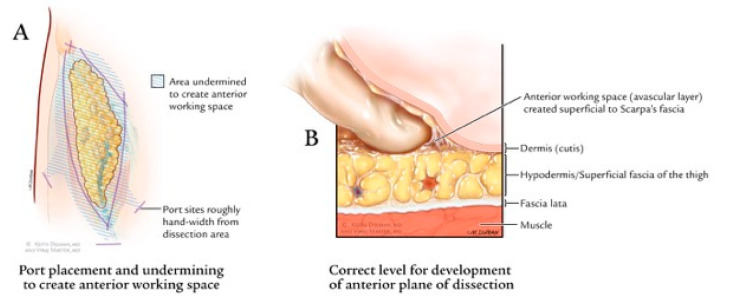
(**a**) Boundaries of dissection as well as area undermined to create anterior working space. (**b**) Correct level for development of anterior plane dissection. Copyright owned by Master et al.

**Figure 4:**
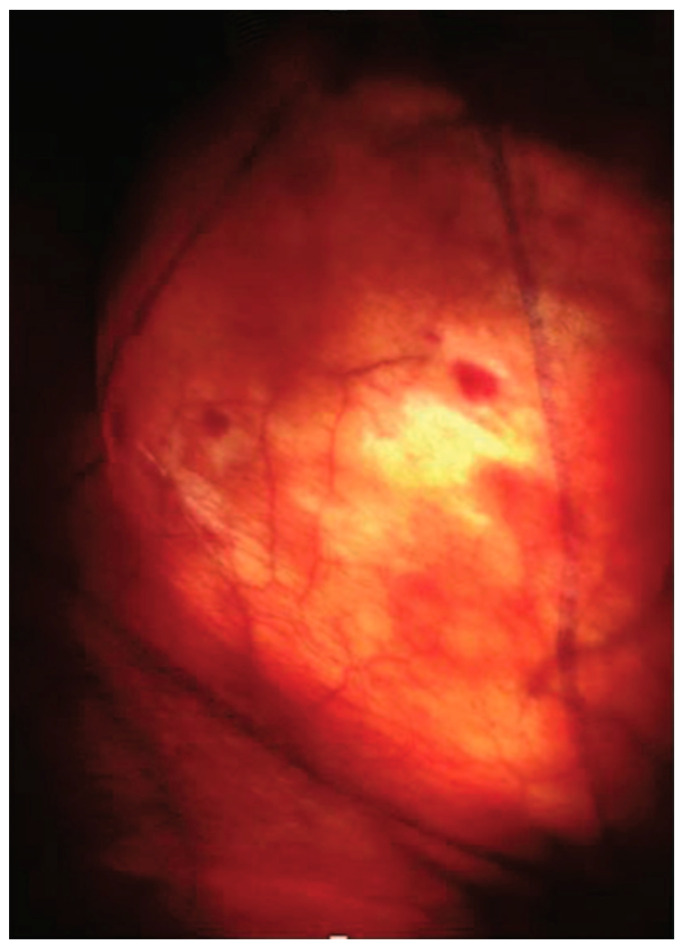
Transillumination of skin flap delineates arterial supply. Copyright owned by Master et al.

The procedure starts by making a 12 mm incision, superficial to Scarpa’s fascia, about 3 cm distal to the apex of the femoral triangle (Figure 2c). Anterior working space is developed which is the area between the skin flaps and the fibrofatty packet containing the lymph nodes (Figure 3). Next, two trocars are inserted approximately a handbreadth from the camera port on each side. Skin flaps are created by dissecting between the Camper’s and Scarpa’s. During this step, transillumination of the skin flaps using laparoscope’s light can show the cutaneous vessels (Figure 4). Care must be taken to not disrupt Camper’s fascia as it can cause damage to the vessels feeding the skin flaps resulting in post-operative skin flap necrosis.

**Figure 5:**
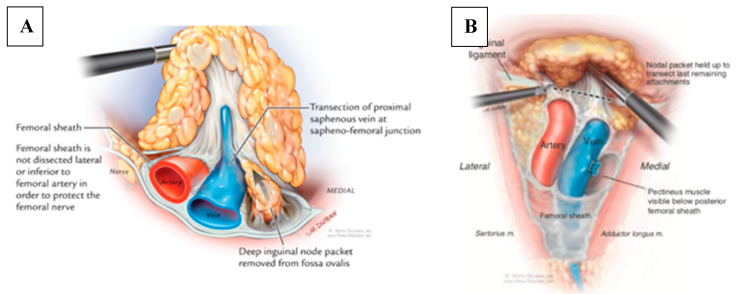
(**a**) Dissection is carried in caudal to cephalad direction. Assistant can lift the nodal packet and allow the surgeon to carry dissection above the vessels. (**b**) All remaining attachments are freed in preparation for nodal retrieving. Copyright owned by Master et al.

**Figure 6:**
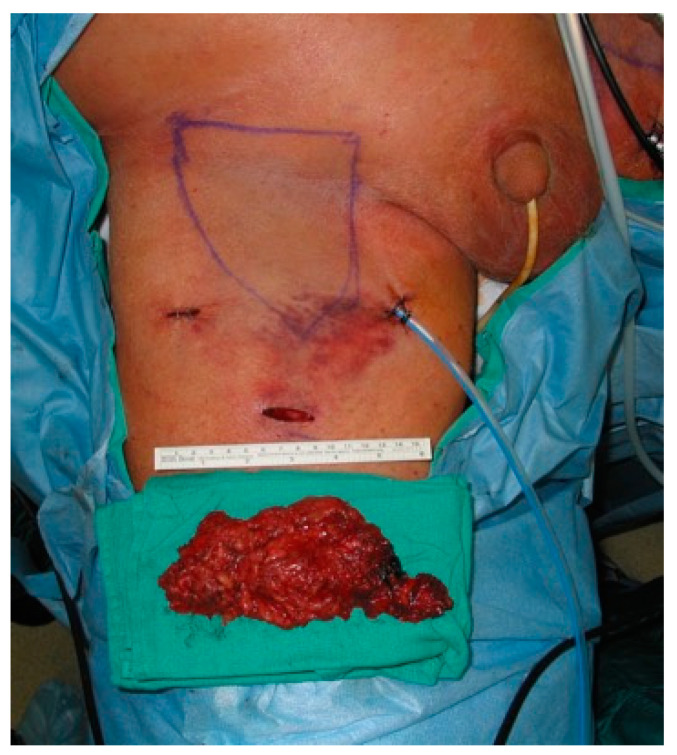
Lymph node packet is retrieved, and a closed-suction drain is placed through one of the port sites. Copyright owned by Master et al.

Next, the anterior working space, which is larger than the dissection boundaries, is developed. Then, dissection of fibrofatty lymph node packet is begun. Lymphatic vessels are sealed with LigaSure or Harmonic device to minimize postoperative lymphorrhea. The saphenous vein is encountered within the apex of the femoral triangle and can be divided with surgical clips or an endovascular stapler. Alternatively, the saphenous vein can be spared if there are no nodes attached to it. Dissection is carried in a caudal to cephalad fashion. Femoral vessels are usually encountered at this stage which are dissected free and skeletonized. The assistant can lift up the packet to provide traction, allowing the surgeon to work below it in the dissection plane (Figure 5a). Dissection is carried to the level of the femoral canal until the pectineus muscle is visualized to ensure complete nodal retrieval, including deep inguinal nodes located on the pectineus muscle. This step also provides exposure for a biopsy of Cloquet’s node if indicated. Finally, the nodal packet is freed up by releasing any remaining attachments to the inguinal ligament (Figure 5b). At the conclusion of the case, lymph nodes are retrieved, and a negative-pressure suction drain is placed through one of the port sites (Figure 6) and the skin incisions are closed. Importantly, simultaneous dissection on both legs can be done by two surgeons, minimizing operative time [22].

### 3.5. Robotic Videoscopic Inguinal Lymphadenectomy (RVEIL) Technique

Robotic-assisted approaches can offer advantages such as three-dimensional views, greater magnification of tissues, and more dexterity. However, utilization of these platforms can increase the cost for the patient and health care system. To date, there is no data to suggest robotic-assisted approach for ILND is superior to video-endoscopic techniques. Decision about which minimally-invasive technique to select mostly depends on availability, surgeons’ experience, patients’ factors, as well as preoperative informed discussion with the patient. Additionally, with robotic-assisted approach the surgeon must be able to trust the bed-side assistant for the assessment of skin flaps during the surgery as he or she is not scrubbed in during the dissection part.

Da Vinci Si and Xi (Intuitive Surgical, Sunnyvale, CA, USA) are the most commonly used platforms for this surgery. In this technique, the patient is placed in supine with legs in frog-leg position (flexion at the knee, with external rotation and abduction of the hip). Approximately 25 cm inferior to the midpoint of the inguinal ligament, an incision is made. This allows the surgeon to properly develop the plane above Scarpa’s fascia. The camera port is placed in this incision and the site is insufflated. The left and right arm ports are placed 6–8 cm medially and laterally to the camera port, outside the inguinal triangle. A third arm is usually not used due to confined anatomical space. Instead an assistant port is usually placed midway between the lateral robotic port and camera port. The boundaries of the dissection are the same as VEIL. 

The superficial dissection is performed by separating the lymph node packet from the fascia lata. Throughout the dissection, encountered lymphatic channels should be done controlled, using cautery or clips, to decrease post-operative lymphatic leakage. After the packet is dissected free from the skin and subcutaneous tissue, it is separated from the inguinal ligament. Deep node dissection involves opening of the fascia lata and dissecting the lymph nodes that are located on the pectineus muscle. Dissection is carried around the femoral vessels, down to the level of the femoral canal. The fossa ovalis is identified and the small branches of the femoral vein and artery are clipped. The saphenous vein may be preserved or ligated. After completion of dissection, the robot is undocked. Specimens, previously placed in a specimen retrieval bag, are removed. Insufflation is decreased under direct visualization to ensure excellent hemostasis has been achieved and a negative-pressure suction drain is placed through one of the port sites. If indicated, the dissection is then performed on the contralateral side in a similar fashion. Current data do not reveal any apparent benefit for targeted lymphatic ligation using chemical agents such as methylene blue. Currently, there is no data on using indocyanine green (ICG) dye with Firefly® fluorescence imaging for targeted lymphatic ligation during ILND.

### 3.6. Post-Operative Care

Postoperative care after ILND differs depending on variables such as the surgical approach and patients’ factors and it also differs from surgeon to surgeon. Convalescence after open ILND, usually involves a period of bed rest and restricted activities of the lower extremities. In contrast, after minimally invasive ILND most surgeons allow for same day ambulation and possible next-day discharge.

Currently, there is no enhanced recovery after surgery (ERAS) protocol or other widely accepted post-operative guidelines. At our institution, we allow post-operative ambulation after minimally-invasive ILND. We use a negative suction drain which is kept for 2–4 weeks and is then removed if output is less than 30 mL/day after ambulation. Patients are advised to wear thigh-high ted hose for 1 month after surgery.

### 3.7. Peri-Operative Outcomes and Complications

Peri-operative outcomes for VEIL, RVEIL and open ILND are summarized in Table A1. Spiess and colleagues presented a review of the literature describing complications and outcomes of open ILND for penile cancer [7]. The authors describe a traditional complication rate of 80–100% predominantly consisting of infection, wound dehiscence/necrosis, and lymphedema. More contemporary series however reported a complication rate of 42–57%. Spiess et al. concluded that the morbidity of open ILND has declined likely due to a combination of surgical technique modifications, improvements in peri-operative care, and patient selection but may have reached a plateau, emphasizing the role for minimally-invasive techniques in this arena [7]. Discussion of the results of minimally invasive techniques is described within this context with published direct comparisons provided where available.

The feasibility and complication rate of minimally invasive techniques have been evaluated in multiple studies. Intraoperative complications are generally rare while done by an expert surgeon. There are two studies that each reported one case requiring conversion from a minimally invasive to open ILND [20,23]. Russell and colleagues described one case complicated by intraoperative bleeding during VEIL requiring blood transfusion and conversion to open approach for bleeding control [20]. The second conversion to open ILND was due to loss of adequate visualization due to dissection deep to the sartorius fascia during RVEIL [23].

Estimated blood loss during RVEIL in different studies ranges from 10–200 mL and appears to be comparable to open ILND as reported by Singh and colleagues [21,23]. Russell and colleagues reported a median blood loss of 50 mL per groin in 27 RVEIL groin dissections [20]. Among seven VEIL groin dissections, Russel et al. estimated a median blood loss of 50 mL per groin. Another study by Wang and colleagues reported a mean blood loss of 22.50 mL during 19 VEIL cases which was significantly less than the mean 68.44 mL blood loss in their 21 open INLD cases (*p*-value = 0.00) [24]. These findings seem to suggest blood loss during VEIL seems to be comparable to that of RVEIL and not worse than traditional open INLD. 

In terms of operative time, while some studies reported no statistically significant difference between minimally-invasive and open ILND [24,25], most papers tend to report a longer operative time for minimally-invasive approaches. Schwentner et al. reported that VEIL took on average 35 minutes longer compared to open ILND (136 vs. 101 minutes, *p* < 0.05) [26]. Similarly, Singh et al. described a longer operative time for RVEIL compared to open approach (75 vs. 60 minutes, *p* < 0.05) [21].

Convalescence is usually faster, less morbid, and better tolerated after minimally-invasive approaches. Koifman et al. retrospectively examined the results of 170 patients undergoing bilateral open ILND, reporting a mean hospital stay of 6.4 days (range 4–27 days) [18]. The reported length of hospital stay after minimally-invasive ILND is variable and ranges from 0–7 days with most authors reporting an average of 1–2 days, which compares favorably to open ILND [27,28]. Singh et al. reported decreased length of hospital stay (3 vs. 4 days; *p* < 0.01) and days to drain removal (12 vs. 15 days; *p* < 0.01) favoring RVEIL compared to open ILND [21].

Post-operative complication rates appear to be substantially decreased in patients treated with minimally-invasive techniques. When comparing VEIL to open ILND, Tobias-Machado and colleagues described a significant decrease in wound complication (0% vs. 50% *p* = 0.02) as well as a trend toward less overall complication rates (20% vs. 70% *p* = 0.06), concordant with findings reported by other investigators [24,25,29]. Similarly, Singh et al. reported lower complication rates with RVEIL compared to open ILND (2% vs. 17%, *p* < 0.05). Of note, the difference in the rate of complications between different series might partially be due to different definitions of complication used by different authors. However, within each study and using the same definition, minimally-invasive techniques appear to have less of both minor and major complications.

In summary, minimally invasive ILND appears to be safe with a decreased overall complication rate and reduced length of stay when compared to open ILND. Blood loss appears to be equivalent between minimally invasive and open techniques while operative time is longer with minimally invasive ILND.

## 4. Oncological Outcomes

Inguinal node metastasis is an important prognostic indicator of survival in penile cancer patients. Additionally, in some patients, ILND appears to offer a survival benefit. When possible, indicated ILND should not be delayed. Kroon and colleagues retrospectively evaluated 40 patients with T2–T3 penile cancer. All patients initially presented with bilateral impalpable lymph nodes. Early ILND was performed when non-palpable inguinal metastasis was detected on dynamic sentinel node biopsy while the delayed group received ILND after nodes became palpable on physical exam during surveillance. Patients undergoing early ILND demonstrated an 85% three-year survival vs. 35% in the delayed group [5].

The oncologic benefits of ILND, especially early ILND, are well established and ILND is currently a core component of multiple guidelines. However, sometimes patients and even providers are reluctant about ILND due to morbidity and high rates of complications. Minimally-invasive technologies seem to result in faster recovery and less complication rate; factors that can make the surgery more appealing for both the patients and providers. Most importantly, how do these approaches compare when oncologic outcomes are considered? 

Nodal yield is routinely used as a surrogate for ILND’s oncological adequacy. In a review of the Surveillance, Epidemiology and End Results (SEER) Program, patients with grade 3 penile cancer that had eight or more lymph nodes removed had a five-year survival of 66.3% compared to 49.2% for those with less than eight nodes removed (*p* = 0.01) [30]. Although there is no single accepted cut-off for number of nodes resected, this study seems to suggest surgeons should aim to obtain at least eight nodes on each side. 

Table A1 contains a summary of lymph node yield from RVEIL, VEIL, and open ILND studies. The majority of current data suggest that minimally-invasive techniques have similar nodal yields compared to traditional open ILND. In a prospective study with 19 RVEIL dissections in 10 patients, the average number of nodes harvested using RVEIL per groin was nine (range 5–21). In this study, at the conclusion of the RVEIL, a separate surgeon examined the operative field for adequacy of the inguinal dissection through a small open incision. The verifying surgeon, using open approach, determined that in 18 of the 19 groin dissections (94.7%) RVEIL was adequate. In this series, two patients were found to have inguinal metastases and all positive nodes were able to be detected and retrieved by RVEIL [23]. Similarly, Singh et al. reported no significant difference in the median number of harvested inguinal lymph nodes when comparing open ILND and RVEIL in their cohort of 51 patients (12.5 vs. 13, *p* = 0.44; range 10.5–14.25 vs. 11–14.5) [21]. 

When it comes to direct comparison between VEIL and RVEIL, the available data are limited. A retrospective study by Russell and colleagues compared 27 RVEIL with seven VEIL cases. The median number of inguinal nodes harvested in VEIL was 10 (range 7.5–12) and 8 in RVEIL (range 6.0–12) respectively. However, this difference was not statistically significant (*p* = 0.84). The small sample size and lack of randomization to treatment group in this study limit our ability to draw a clear conclusion [20].

In addition to the nodal yield, local tumor recurrence after ILND is another important end point to consider. Inguinal recurrence of penile cancer following primary tumor resection and inguinal lymphadenectomy is estimated to be about 16% in node positive patients at five years with a median time to recurrence of 5.3 months [31,32]. A review by Hu et al. analyzed 10 randomized control trials that compared local recurrence rate between VEIL and open INLD. This study reported no significant difference in recurrence rate between open INLD and VEIL approach (odds ratio (OR) 1.54, 95% confidence interval (CI) 0.41–5.84) [33]. In their cohort of 51 patients, Singh et al. reported no inguinal recurrences in either the RVEIL or open ILND group cohort with a median follow-up of 40 months. In this study, 21 of the 51 RVEIL patients (41.2%) and 32 of the 100 open ILND patients (32%) had positive lymph nodes [21]. If adjuvant radiotherapy is being considered in the setting of positive nodes, the radiation field can be extended to include the port sites to reduce the theoretical risk of port-site metastasis.

Multiple single-institutional case-control series have evaluated the oncological adequacy of VEIL and RVEIL, using retrieved nodal count and inguinal recurrence as measures for oncologic quality. These studies appear to suggest that minimally-invasive techniques can lead to similar oncologic outcomes when compared to open technique. Utility of minimally-invasive ILND is promising and merits further investigation by larger randomized trials with longer follow-ups.

Finally, minimally-invasive techniques would theoretically allow physicians to deliver more timely adjuvant therapies in patients at high risk of relapse due to lower risk of wound healing complications and shorter recovery time. Although data are lacking in the literature and prospective studies are needed, a more widespread use of minimally-invasive ILND yields the potential to revitalize the multidisciplinary collaboration among specialists to allow the administration of combined modality therapy protocols and improve outcomes.

## 5. Conclusions

Inguinal lymph node dissection has an important role in both staging and treatment of penile cancer. Minimally-invasive techniques in penile cancer patients with clinical stage N0–N2 disease can reduce the morbidity and complications associated with ILND. As a result, patients can tolerate this procedure better and surgeons can offer surgery to patients who otherwise would not be a candidate or personally willing to undergo surgery. Importantly, minimally-invasive approaches appear to yield similar short to mid-term oncological outcomes as open techniques.

## Appendix A

**Table A1:** Summary of Peri-operative Outcomes for Video Endoscopic Inguinal Lymphadenectomy (VEIL), Robotic-assisted Video Endoscopic Inguinal Lymphadenectomy (RVEIL) and Open Inguinal Lymphadenectomy (OILND) Studies.

Author, (Year)	*n* of Patients (*n* of VEIL)	Histology	Blood Loss (mL)	Lymph Node Yield; Positive Nodes	Drain Removal in Days	Hospital Days	Post-Op Complications
**VEIL Studies**							
Wang et al. (2017) [24]	16 (19)	Penile cancer	Mean: 22.50; SD: 14.24	Mean: 10.78 SD: 5.22; 11 positive nodes	Mean: 7.23; SD: 1.79	Mean: 10.43; SD: 2.53	Prolonged lymphedema 15.8% Wound infection 5.3% Skin necrosis 5.3%
Kumar and Sethia, (2017) [25]	20 (33)	Penile cancer	n/a	Mean: 9.36; Mean Positive nodes: 1.24	n/a	Mean: 2.5; Range: 0–14	Wound complications 6% Prolonged lymphedema 3% Lymphocele 27%
Schwentner et al. (2013) [26]	16 (28)	Penile: 14 patients Melanoma: 2 patients	n/a	Mean: 7.1; Range: 4–13; positive nodes Mean: 1.6 SD: 1.9	n/a	n/a	Lymphatic complication 7.1% Post-operative hemorrhage 7.1%
Tobias-Machado et al. (2008) [29]	15 (20)	Penile SCC	n/a	Mean: 10.8; Range: 7–16; 4 positive nodes	Mean: 4.9; Range: 3–12	**B/L VEIL** Mean: 24 h; Range: 12–36 h **VEIL/Open** Mean: 6.4 days; Range: 5–10 days	Total: 20% Hematoma 5% Lymphocele 5% Lymphorrhea 5% Skin necrosis 5%
**RVEIL Studies**							
Singh et al. (2018) [21]	51 (102)	Penile SCC	n/a	Median: 13; IQR: 11–14.5; 21 patients with positive nodes	Median: 12	Median: 3	Total major: 2% Edge necrosis 9.8% Flap necrosis 2%
Russel et al. (2017) [20]	14 (27)	Penile cancer	Median: 50.0 IQR 15.0–50.0	Median: 8; IQR: 6–12; 22% of limbs had positive superficial LN 20% of limbs had positive deep LN	Median: 36.0; IQR: 24–48.5	Median: 1; Range: 1–2	Total: 21% Lymphocele 4% Cellulitis 4% Abscess 4%
Alhawat et al. (2016) [19]	3 (6)	2 Penile cancer, 1 Urethral cancer	Mean: 66.66; Range: 50–80	Mean: left 18, right 14.6; 19 positive nodes, 3 patients	Mean: 44.6; Range: 34–72	3	Total: 33.3% Lymphocele 33.3%
Matin et al. (2013) [23]	10 (20)	Penile cancer	Median: 100; Range: 10–200	**Left** Mean: 9; Range: 5–21 **Right** Mean: 9; Range: 6–17 11 positive nodes (2 patients)	n/a	n/a	Total: 60% Open conversion 20% Skin necrosis 10% Wound breakdown 10% Cellulitis 20% Abscess 10%
**Open ILND**							
Tsaur et al. (2015) [15]	29 (57 limited inguinal lymph node dissections)	Penile cancer	n/a	Mean: 8.1; SD: 3.7	n/a	Mean: 14.2; SD: 6.1	Total rate: 54% 15 major and 16 minor complications Wound infection 22.8% Leg edema 15.8% Seroma/lymphocele 10.5% Hematoma 1.8% Paresthesia 1.8% DVT 1.8%
Gopman et al. (2014) [17]	327 (374 modified inguinal lymph node dissections)	Penile cancer	n/a	**Left** Mean: 7; Range: 0–26 Mean positive: 1; Range: 0–9 **Right** Mean: 7; Range: 0–24 Mean positive: 1; Range: 0–10	Mean: 11; Range: 0–61	Mean: 9.6; Range: 0–62	Total: 55.4% of patients Major: 34.3% Minor: 65.7%
Koifman et al. (2013) [18]	170 (340)	Penile cancer	n/a	Per groin: Mean: 10.9; Range: 6–19	n/a	Mean: 6.4; Range: 4–27	Total: 10.3% 10 major and 25 minor complications Lymphedema 4.1% Seroma 1.2% Scrotal edema 0.9% Skin edge necrosis 0.9% Lymphocele 0.9% Wound infection 0.6% Wound abscess 0.6% DVT 0.6%
Yao et al. (2009) [16]	75 (150 modified inguinal lymph node dissections)	Penile cancer	n/a	n/a; 52% node positivity rate	n/a	n/a	Total: 37 complications Wound infection 1.4% Skin necrosis 4.7% Lymphedema 13.9% Lymphocele 2% DVT 0.7%
Kroon et al. (2005) [5]	40 (*n*/A); 20 patients underwent resection upon discovery of palpable nodes during physical exam (delayed), 20 underwent DSNB prior to development of palpable nodes (early)	Penile cancer (T2–3)	n/a	n/a; node positivity per groin: **Early Group** Mean: 1.6; Range: 1–5 **Delayed Group** Mean: 2.1; Range: 1–6	n/a	n/a	n/a
